# TNF-α and RPLP0 drive the apoptosis of endothelial cells and increase susceptibility to high-altitude pulmonary edema

**DOI:** 10.1007/s10495-024-02005-9

**Published:** 2024-08-07

**Authors:** Yi-Ling Ge, Pei-Jie Li, Ying-Rui Bu, Bin Zhang, Jin Xu, Si-Yuan He, Qing-Lin Cao, Yun-Gang Bai, Jin Ma, Lin Zhang, Jie Zhou, Man-Jiang Xie

**Affiliations:** 1 Department of Aerospace Physiology, Key Laboratory of Aerospace Medicine of Ministry of Education, Fourth Military Medical University, Xi’an, Shaanxi Province 710032 China; 2 Department of Endocrinology, Xijing Hospital, Air Force Medical University, No. 127 Changle West Road, Xi’an, 710032 China

**Keywords:** High-altitude pulmonary edema, Acclimatization, Adaptation, Apoptosis, Bioinformatic analysis

## Abstract

High-altitude pulmonary edema (HAPE) is a fatal threat for sojourners who ascend rapidly without sufficient acclimatization. Acclimatized sojourners and adapted natives are both insensitive to HAPE but have different physiological traits and molecular bases. In this study, based on GSE52209, the gene expression profiles of HAPE patients were compared with those of acclimatized sojourners and adapted natives, with the common and divergent differentially expressed genes (DEGs) and their hub genes identified, respectively. Bioinformatic methodologies for functional enrichment analysis, immune infiltration, diagnostic model construction, competing endogenous RNA (ceRNA) analysis and drug prediction were performed to detect potential biological functions and molecular mechanisms. Next, an array of in vivo experiments in a HAPE rat model and in vitro experiments in HUVECs were conducted to verify the results of the bioinformatic analysis. The enriched pathways of DEGs and immune landscapes for HAPE were significantly different between sojourners and natives, and the common DEGs were enriched mainly in the pathways of development and immunity. Nomograms revealed that the upregulation of TNF-α and downregulation of RPLP0 exhibited high diagnostic efficiency for HAPE in both sojourners and natives, which was further validated in the HAPE rat model. The addition of TNF-α and RPLP0 knockdown activated apoptosis signaling in endothelial cells (ECs) and enhanced endothelial permeability. In conclusion, TNF-α and RPLP0 are shared biomarkers and molecular bases for HAPE susceptibility during the acclimatization/adaptation/maladaptation processes in sojourners and natives, inspiring new ideas for predicting and treating HAPE.

**Supplementary Information:**

The online version contains supplementary material available at 10.1007/s10495-024-02005-9.

## Introduction

Thousands of people travel to altitude regions with elevations > 2500 m for recreational, economic, military or religious purposes every year [1]. However, as altitude increases, the progressively reduced inspired oxygen pressure induces hypoxic stress and triggers high-altitude illnesses. High-altitude pulmonary edema (HAPE) is a potentially lethal manifestation of high-altitude illness that usually occurs at any point 1–5 days following acute exposure to high-altitude hypoxia. HAPE is a noncardiogenic but high-permeability alveolar edema [1], in which extremely thin blood-gas barriers are not protected from uneven vasoconstriction-induced high capillary pressure and develop ultrastructural and mechanical disruptions with high-molecular-weight protein leakage [2, 3]. HAPE incidence is closely associated with the rate of ascent and altitude reached [4]. In addition, epidemiology studies have demonstrated that HAPE has individual susceptibility with race-specific and family-specific tendencies [5]. For example, native Tibetans have a much lower HAPE incidence than immigrant low-altitude populations, and patients with a family history of HAPE have a high risk of HAPE recourrence [5].

To defend against oxygen deprivation at high altitude, a complex and finely integrated physiological process termed acclimatization, which usually comprises cardiopulmonary and hematological responses, including hyperventilation, hemoconcentration, and increased heart rate and cardiac output, is mobilized in sojourners [6]. Sojourners with efficient acclimatization can tolerate and adapt to the hypobaric hypoxic environment and are usually insusceptible to high-altitude illnesses [3]. In addition, over 150 million people worldwide have inhabited areas at altitudes > 3000 m for hundreds of generations [7]. High-altitude natives have developed many distinctive patterns and biological characteristics termed adaptation, including augmented aerobic performance with increased lung volume and capacity, increased redox status and nitric oxide metabolites, and attenuated maladaptive responses of hypoxic pulmonary vasoconstriction and erythrocytosis to adapt to oxygen deficiency [3, 8–10]. Notably, the processes and mechanisms of acclimatization in sojourners and adaptation in natives are different, which involve genetic variations in protein activity, organelle function, cellular metabolism, and organ plasticity, but both help individuals offset the adverse effects of hypobaric hypoxia and reduce the incidence of high-altitude illnesses at high altitudes [8, 10, 11]. Thus, comparing HAPE patients with acclimatized sojourners and adapted natives to distinguish the different molecular bases of acclimatization and adaptation may be beneficial for gaining a better understanding of HAPE susceptibility.

In recent years, genetic evidence for high-altitude illnesses has emerged, and comparisons of genetic profiles between high-altitude natives and lowlanders have revealed that EPAS1, EGLN1 and PPARA are strong candidate genes for natural selection at high altitudes [3, 9, 10]. There is much interest in clarifying the genetic factors and molecular basis of HAPE susceptibility and pathogenesis, but no definitive gene differences or specific biomarkers for HAPE susceptibility have been reported thus far [3, 12, 13]. The rapid development of sequencing technologies has led to new approaches. In the present study, the GSE52209 dataset was selected to explore the blood expression profiles of HAPE patients versus acclimatized sojourners and adapted natives from Ladakh as controls. In addition, Venn analysis revealed the common and disparate differentially expressed genes (DEGs) involved in the pathogenesis of HAPE between acclimatized sojourners and adapted natives, and a set of bioinformatic methodologies and experimental validations were applied to identify the hub genes and explore their potential biological functions and molecular mechanisms. By separating the distinctive transcript features of adaptation and acclimatization processes, our study provides a novel perspective for understanding HAPE pathogenesis and finding specific biomarkers for HAPE susceptibility, which may inspire new ideas for predicting and treating HAPE.

## Materials and methods

### Datasets

The GPL9365 platform is an Ocimum Biosolutions Human 40k OciChip consisting of 20,160 spots printed on Corning^®^ epoxy-coated glass slides using Omnigrid (Gene Machines). The GSE52209 dataset generated on the GPL9365 platform was obtained from the Gene Expression Omnibus (GEO) database (https://www.ncbi.nlm.nih.gov/geo/) and integrated into our analysis. This dataset contains blood samples from 14 sojourners who were acclimatized to high altitude and were grouped as controls, 14 high-altitude natives from Ladakh (HAN) and 17 individuals who developed high-altitude pulmonary edema within 48–72 h after air induction to high altitude (HAPE).

### Differentially expressed gene (DEG) analysis

Microarray data from the GEO database were retrieved and processed using the “GEOquery” package (version 2.66.0). DEG analysis was conducted using the “limma” package (version 3.54.0) according to the criteria of adjusted *p* value < 0.05 and absolute[log2FC] > 1. The “ggplot2” (version 3.4.0) and “ComplexHeatmap” (version 2.14.0) packages were used to visualize and generate volcano plots and heatmaps, respectively.

### Functional enrichment analysis

To explore the biological significance of the DEGs, Gene Ontology (GO) and Kyoto Encyclopedia of Genes and Genomes (KEGG) pathway enrichment analyses were performed using the “clusterProfiler” package (version 4.2.2). An adjusted *p* value < 0.05 was considered significant.

### Immune infiltration analysis

xCell is a novel and validated gene signature-based strategy used to infer immune and stromal cell types [14]. The infiltration levels of various immune cell types in the HAPE group were evaluated using xCell and compared with those in the control and HAN groups in GSE52209. Group boxplots were generated and compared using the Wilcoxon rank-sum test to determine significant differences between cell types, with a cutoff adjusted *p* value < 0.05.

### Construction of a protein–protein interaction (PPI) network and selection of hub genes

The common/divergent DEGs were classified and marked as gene sets of A (altered only between HAPE and Control groups), B (altered only between HAPE and HAN groups) and C (altered in the HAPE group as compared with both Control and HAN groups). The protein–protein interaction (PPI) pairs of DEGs in the A/B/C gene sets were investigated online using the STRING database (http://string-db.org), and the PPI networks were visualized using Cytoscape (version 3.6.1). Five algorithms, namely, maximal clique centrality (MCC), maximum neighborhood component (MNC), edge percolated component (EPC), degree and bottleneck, were subsequently used to calculate each gene. The top 10 genes in each algorithm were further overlapped using Venn analysis and identified as hub genes.

### Nomogram construction and receiver operating characteristic (ROC) curve evaluation

To predict the probability of HAPE from the expression of the hub genes, nomogram models were constructed using the “rms” package. A calibration curve was plotted to assess the predictive ability of the nomograms, in which the Hosmer–Lemeshow (HL) test was used to determine the degree of dispersion between the predicted value and true value (*p* value > 0.05 suggested that there was no difference between the predicted value and true value and that the model had excellent goodness-of-fit). In addition, to assess the diagnostic ability of the nomograms, receiver operating characteristic (ROC) curves were constructed using the “pROC” package, and the area under the curve (AUC) was calculated, with values closer to 1 suggesting greater diagnostic accuracy.

### Construction of competing endogenous RNAs (ceRNA networks)

The ElMMo database (http://www.mirz.unibas.ch/ElMMo3/) is a Bayesian target prediction algorithm that can automatically infer the functional sites and putative target sites of each miRNA and hence was used to predict the mRNA-miRNA interaction pairs targeting hub genes in the present study [15]. Then, based on the top 5 miRNAs with the highest binding scores, the starBase database was used to screen for miRNA‒lncRNA interactions and finally construct a ceRNA network of mRNAs-miRNAs-lncRNAs. Cytoscape was used to visualize the ceRNA network.

### Drug and compound prediction

The Drug Signatures Database (DSigDB, http://dsigdb.tanlab.org/DSigDBv1.0/) was used to predict the drugs that potentially act on the hub genes according to the criterion of an adjusted *p* value < 0.01. The Encyclopedia of Traditional Chinese Medicine (ETCM; http://www.nrc.ac.cn:9090/ETCM/) database was used to predict the traditional Chinese medicines (TCMs) that potentially act on the hub genes. Then, according to the threshold of bioavailability ≥ 30% and drug likeness ≥ 0.18, the Traditional Chinese Medicine Systems Pharmacology Database and Analysis Platform (TCMSP; https://www.tcmsp-e.com/) was used to screen the active ingredients and effective components of the predicted TCMs. Cytoscape was used to visualize the networks of Drugs-Genes and TCMs-ingredients-Genes.

### HAPE rat model

Adult male SD rats (10–12 weeks old, body weight > 300 g) were used in this study. All the rats were raised in specific pathogen-free (SPF) cages at a suitable temperature (25 ± 5 °C) and humidity (50 ± 5%). All experiments involving animals were approved by the Animal Care and Use Ethics Committee of Air Force Medical University.

The HAPE rat model was established based on a previously described method [16]. However, in the present study, we found 3 days is the most appropriate time for acute modeling, which causes the most severe lung injury. The hypobaric chamber system was developed by our laboratory to mimic the high-altitude environment and consists of a vacuum pump, a control panel, a pressure and flow control/monitoring probe and hypobaric chambers [17]. The rats were exposed to a simulated altitude of 6000 m (the barometric pressure is about 353.88 mmHg and oxygen partial pressure is about 74.0 mmHg), with a height increase rate of 20 m/s, and the temperature and humidity were maintained at 25 °C and 55%, respectively.

### Wet/dry weight ratios of the lung tissues

Following the euthanasia of rats, wet weight was measured immediately after the middle lobe of right lung being excised. Thereafter, the lung tissues were dried at 60℃ in a constant-temperature drying oven for 48 h and weighed again to determine the dry weight. Finally, the wet/dry (W/D) weight ratio of the lung tissues was calculated.

### Hematoxylin-eosin (H&E) and immunofluorescence staining

After the middle lobe of right lung was collected, the lung tissues were fixed with 4% paraformaldehyde for 24 h. After the paraffin sections were prepared, dewaxing, hematoxylin staining, eosin staining, and dehydration were sequentially performed. Images were captured using photo-microscopy (Olympus, Tokyo, Japan).

Rat lung tissues in paraffin-embedded sections were deparaffinized, rehydrated, fixed, blocked with 5% BSA, and then incubated with primary antibodies against cleaved caspase-3 at a 1:200 dilution (#9661; Cell Signaling Technology, USA), RPLP0 at a 1:200 dilution (11290-2-AP; Proteintech, China) and CD31 at a 1:500 dilution (28083-1-AP; Proteintech, China) at 4 °C overnight, followed by incubation with fluorescently labeled secondary antibodies. Then, 1 µg/mL Hoechst 33,258 solution (Thermo Fisher Scientific, MA, USA) was used for nuclear staining. The stained sections were examined using Pannoramic MIDI and evaluated using Pannoramic Viewer (3DHISTECH, Budapest, Hungary).

### Transmission electron microscopy (TEM)

A portion of the inferior lobe tissue of the right lung was removed, immediately fixed with 4% glutaraldehyde (Servicebio, Wuhan, China) and then trimmed into 0.1 × 0.1 × 0.1 cm^3^ blocks for further fixation with 1% osmium tetroxide in deionized water. Subsequent steps were performed as previously described [17]. Electron micrographs were obtained using TEM (HITACHI 7800, Tokyo, Japan) at 80 kV. The ultrastructure of the lung tissues and pulmonary endothelium were observed at ×8k and ×25k magnifications.

### Cell culture and lentiviral transduction

Human umbilical vein endothelial cells (HUVECs) were obtained from ScienCell (Carlsbad, CA, USA) and cultured in Dulbecco’s modified Eagle’s medium supplemented with 10% fetal bovine serum (FBS) (Thermo Scientific, Rockford, IL, USA), 100 U/mL of penicillin (Solarbio, Beijing, China), and 100 µg/mL of streptomycin (Solarbio, Beijing, China).

HUVECs were transduced with a lentivirus carrying RPLP0 short hairpin RNA (shRNA). For lentiviral transduction, shRNAs targeting *RPLP0* were selected with the greatest degree of transcript reduction and subsequently cloned and inserted into PDS278-pL-U6-shRNA-GFP-puro (Sigma‒Aldrich, SHC001) to obtain the corresponding shRNA vectors. A nontargeting shRNA vector (NC shRNA, Sigma-Aldrich, SHC002), was used as a control for lentiviral infection. To produce lentiviral particles, HEK-293T cells were cotransfected with pCMV-dR8.91 dvpr, pCMV-VSV-G, and *RPLP0*-pLKOshRNA with JetPei (Genycell Biotech, 101–05). The supernatant was collected 48 h after transfection and concentrated by ultracentrifugation using Centricon Plus-100 filters (Millipore, 831,826). To generate stable cell lines with downregulated RPLP0 expression, HUVECs were infected with viral particles at a 1:4 dilution in the presence of 8 µg/ml of polybrene (Sigma‒Aldrich, 107,689). After 24 h, the cells harboring the *RPLP0* shRNA or NC shRNA cassette were selected in the presence of puromycin (1.5 µg/ml; Sigma-Aldrich) for 3 d. The expression of the constructs was confirmed by western blot analysis.

### Detection of endothelial permeability

The rats were injected with Evans blue dye (20 mg/mL) through the femoral vein (60 mg/kg). One hour after injection, the vasculature was perfused with saline to remove intravascular Evans blue dye, and the upper lobe tissue of the right lung was collected and homogenized. The protein concentration of the lung tissue homogenate was assessed using the BCA assay. The concentration of Evans blue dye in the lung tissue supernatant was determined by measuring the absorbance at 620 nm and normalizing it to the protein concentration [18].

HUVECs (8 × 10^4^ were seeded into 12-well transwell filters (3-µm pore size; Corning) and then incubated when the cell confluency reached 100%. The 2% Evans blue dye-labeled albumin was diluted to 0.067, 0.0335, 0.01675, 0.0067 and 0.00335 mg/mL, and the absorbances of different concentrations of Evans blue dye-labeled albumin were measured at 620 nm to construct a standard concentration-absorbance curve. For detecting the HUVECs permeability, 0.67 mg/mL Evans blue dye-labeled albumin was added to the upper chamber, and after 1 h of incubation, the lower medium was collected, and the concentration of Evans blue dye was determined by measuring the absorbance at 620 nm and normalized to a standard curve of known Evans blue dye concentrations [19].

### TUNEL staining

The apoptosis of HUVECs and ECs in rat lung tissues was detected using TUNEL staining according to the manufacturer’s instructions using a commercially available kit (HY-K1078; MedChemExpress, USA). The numbers of TUNEL-positive ECs and total cells were counted, and apoptosis was evaluated by the ratio of positive cells to total cells.

### ELISA

The protein levels of TNF-α in the rat serum and lung tissues were measured using ELISA kits according to the manufacturer’s instructions (HEA133Ra; Cloud-Clone Corp, China).

### Western blotting and RT‒qPCR analysis

HUVECs and rat lung tissues were dissociated with lysis buffer, and protein was subsequently extracted and quantified using a BCA kit (Thermo Fisher Scientific, MA, USA). Equivalent amounts of proteins from different groups were loaded, electrophoresed and transferred to a polyvinylidene difluoride membrane (Millipore, Schwalbach, Germany). The membrane was blocked with 5% BSA in Tris-buffered saline with 0.1% Tween 20, followed by incubation with primary antibodies against cleaved caspase-3 at a 1:2000 dilution (#9661; Cell Signaling Technology, USA) and RPLP0 at a 1:1000 dilution (11290-2-AP; Proteintech, China) overnight at 4 °C. The membrane was subsequently incubated with appropriate HRP-linked secondary antibodies for 1.5 h at room temperature. The membrane was finally imaged using an Odyssey scanner (LI-COR Biosciences, NE, USA) and analyzed using NIH ImageJ software.

HUVECs and rat lung tissues were homogenized with RNAiso (Takara, Otsu, Japan). Complementary DNA (cDNA) was amplified with SYBR Premix Ex TaqTM (TaKaRa Bio, Otsu, Japan). RT-qPCR was performed using SYBR Green PCR master mix (Life Technologies, USA) according to the manufacturer’s instructions (95 °C × 15 s, 60 °C × 30 s, 40 cycles), and the fluorescence signals of the melting curve were collected. Each sample was tested three times. Actb was used as the control for PCR product quantification and normalization. The data were analyzed using the relative Ct (2 − ΔΔCt) method and are expressed as the fold change compared with the respective control.

### Statistical analysis

All continuous variables with a normal distribution are presented as the mean ± SEM. Comparisons between 2 groups were performed using 2-tailed unpaired Student’s t test with Welch’s correction. Statistical differences among groups were analyzed by ordinary one-way ANOVA followed by Tukey’s multiple comparison test. Significance in immune infiltration analysis was determined by the Wilcoxon rank-sum test. All the statistical analyses were performed using Prism software (GraphPad Prism for Windows, version 9.0; Nashville, TN). Differences were considered significant at *P < 0.05, **P < 0.01, and ***P < 0.001 [20].

## Results

### Differential gene expression profiles and immune landscapes for HAPE between acclimatized sojourners and adapted natives

**Fig. 1:**
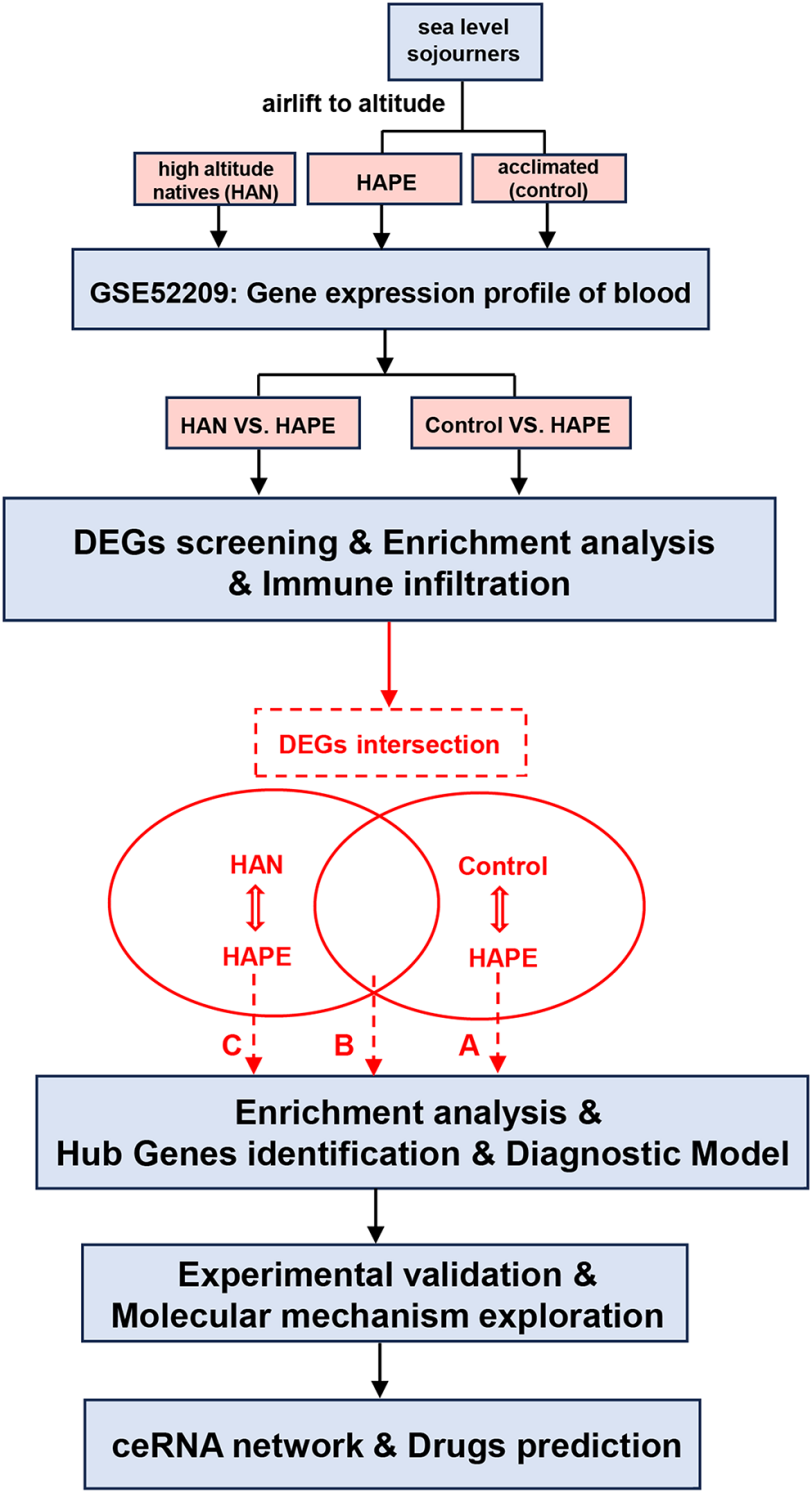
Flowchart of the bioinformatic analysis and validation strategy for GSE52209. The common/divergent DEGs were classified and marked as gene sets of **A** (altered only between HAPE and Control groups), **B** (altered only between HAPE and HAN groups) and **C** (altered in the HAPE group as compared with both Control and HAN groups)

**Fig. 2:**
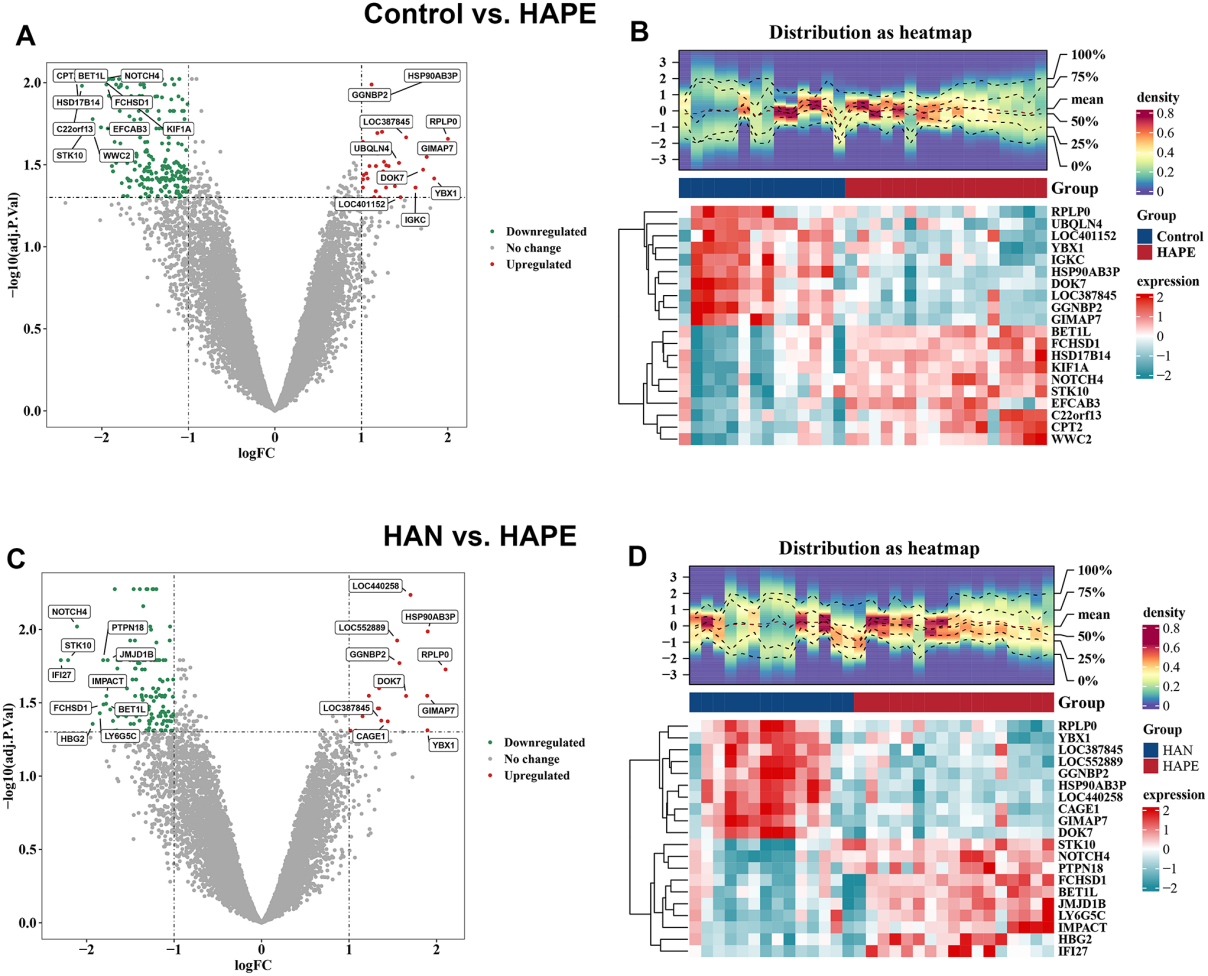
Identification of differentially expressed genes (DEGs) in HAPE patients compared with acclimatized sojourners and adapted natives. **A** and **B**, Volcano plot (**A**) and heatmap (**B**) of differentially expressed genes (DEGs) between the HAPE group and acclimatized sojourner (Control) group. **C** and **D**, Volcano plot (**C**) and heatmap (**D**) of DEGs between the HAPE and adapted native (HAN) groups. Volcano plot: each dot represents a gene, the green dots indicate downregulated genes, the red dots indicate upregulated genes, and the gray dots indicate nonsignificant genes. The names of the top 20 genes with the greatest significant differences are labeled. Heatmap: gene density is shown at the top, and the expression of the top 20 DEGs is shown at the bottom (red represents high expression, and green represents low expression)

**Fig. 3:**
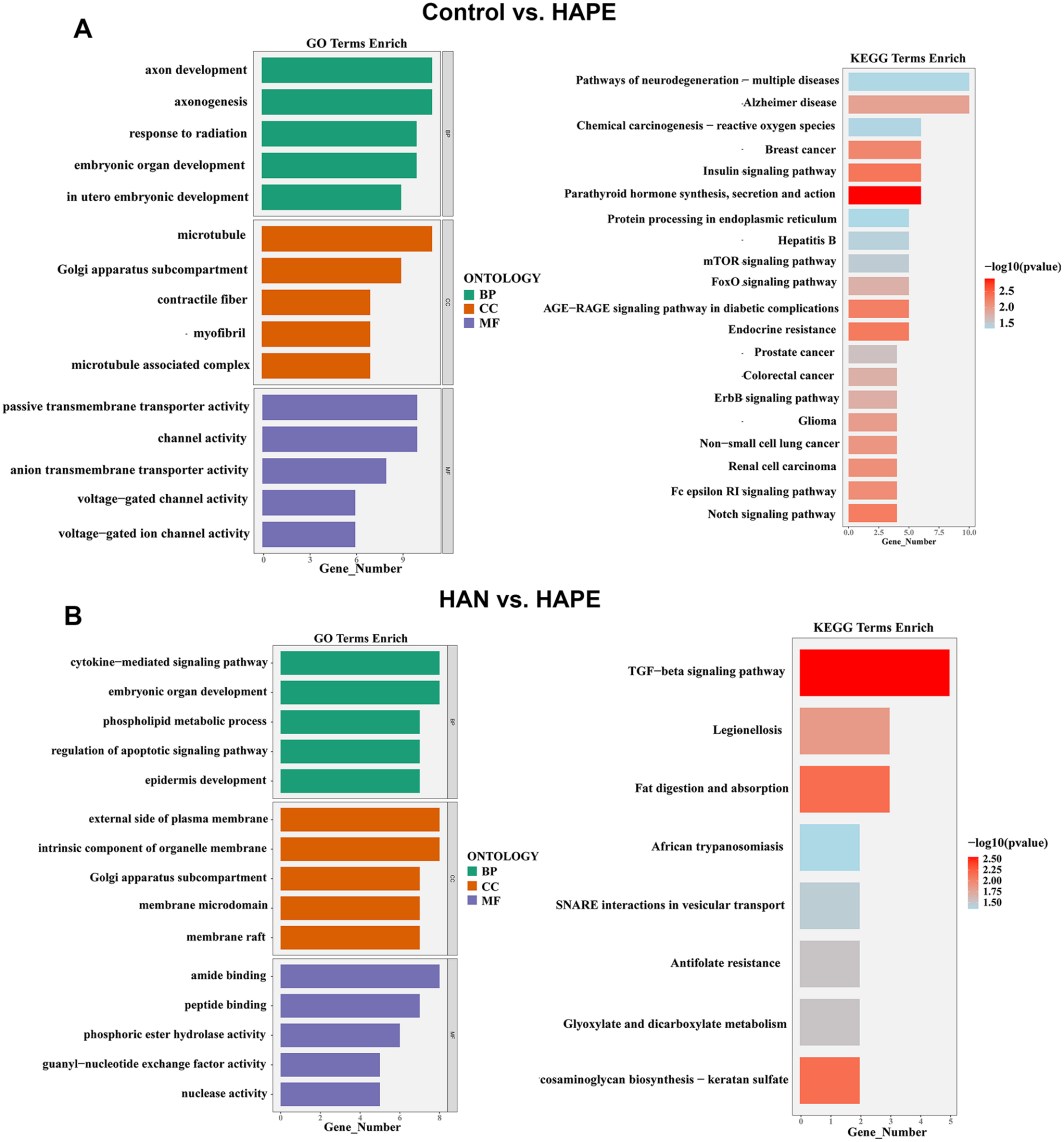
GO and KEGG enrichment analyses of DEGs. **A**, GO and KEGG terms of DEGs between the HAPE and control groups. **B**, GO and KEGG terms of DEGs between the HAPE and HAN groups. The top 5 enriched biological process (BP), cellular component (CC), and molecular function (MF) GO terms and the top 20 KEGG terms are listed

**Fig. 4:**
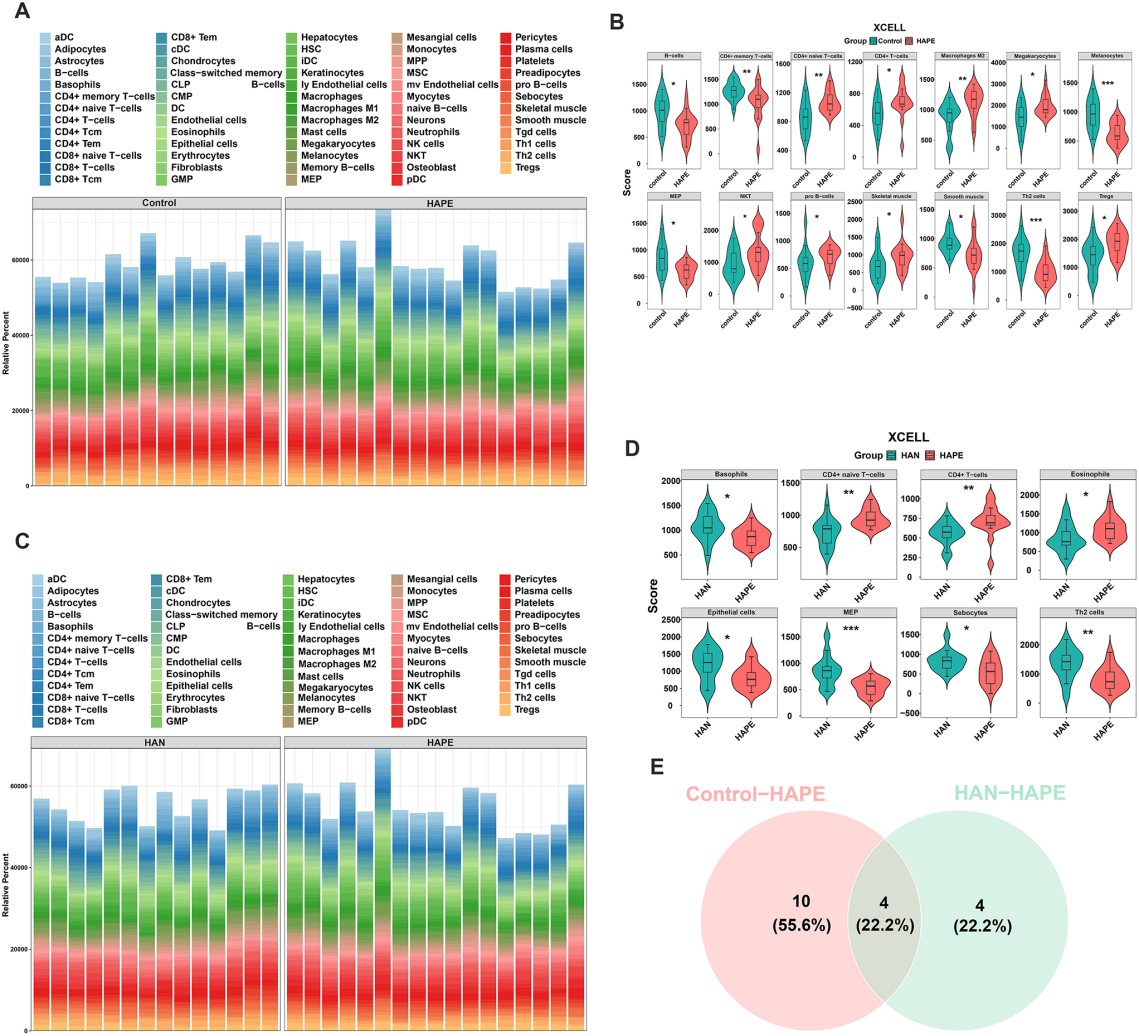
Immune cell landscapes in HAPE patients compared with those in acclimatized sojourners and adapted natives. **A** and **B**, Immune cell landscapes associated with the abundance of 64 immune cells (**A**) and immune cell enrichment scores (**B**) between the HAPE and control groups, as determined using the xCell algorithm. **C** and **D**, Immune cell landscapes associated with the abundance of 64 immune cells (**C**) and immune cell enrichment scores (**D**) between the HAPE and HAN groups, as determined using the xCell algorithm. **E**, Venn diagram showing 4 common immune cells that were altered unanimously in the HAPE group compared with the control and HAN groups. *P < 0.05, **P < 0.01, ***P < 0.001, ****P < 0.0001. Significance was determined by the Wilcoxon rank-sum test (B and D). *P < 0.05, **P < 0.01, *** *P* < 0.001, **** *P* < 0.0001 versus the Control or HAN

The expression profiles of blood samples from sojourners who were acclimatized to high altitude (*n* = 14), high-altitude natives (*n* = 14) and individuals who developed high-altitude pulmonary edema (*n* = 17) were published in the GSE52209 dataset and further analyzed using bioinformatic methodology and experimental validation in our present study (Fig. 1). First, based on the criteria of *P*-adjustment < 0.05 and absolute [log2 FC] > 1, we screened 270 differentially expressed genes (DEGs) between the HAPE and acclimatized sojourner (Control) groups and 166 DEGs between the HAPE and high-altitude native (HAN) groups. Volcano plots and heatmaps of these DEGs are shown, along with the top 20 upregulated and downregulated genes highlighted in Fig. 2A and D. Functional enrichment analysis of these DEGs revealed that, between HAPE patients and acclimatized sojourners, the DEGs were enriched mainly in the GO terms of axon development, transmembrane transporter activity and channel activity and in the KEGG terms of neurodegeneration and Alzheimer’s disease (Fig. 3A), whereas between HAPE patients and adapted natives, the DEGs were enriched mainly in the GO terms of the cytokine-mediated signaling pathway and embryonic organ development and in the KEGG terms of the TGF-beta signaling pathway (Fig. 3B). Next, the xCell algorithm was used to analyze the infiltration of 64 immune cell types. Compared with those of acclimatized sojourners, the abundances of CD4^+^ native T cells, CD4^+^ T cells, M2 macrophages, megakaryocytes, NKT cells, pro-B cells, skeletal muscle cells, smooth muscle cells and Tregs were significantly greater in HAPE patients (Fig. 4A and B). However, compared with those of adapted natives, only three types of immune cells, including CD4^+^ native T cells, CD4^+^ T cells and eosinophils, were significantly greater in HAPE patients (Fig. 4C and D). Among these HAPE-related immune cells, Venn diagrams revealed 4 common immune cell types, CD4^+^ native T cells, CD4^+^ T cells, MEPs and Th2 cells, which were altered unanimously in the sojourners and natives (Fig. 4E). These results suggest that the expression profile and immune landscape of HAPE differ between sojourners and natives.

### Screening and functional analysis of common/divergent DEGs for HAPE between acclimatized sojourners and adapted natives

**Fig. 5:**
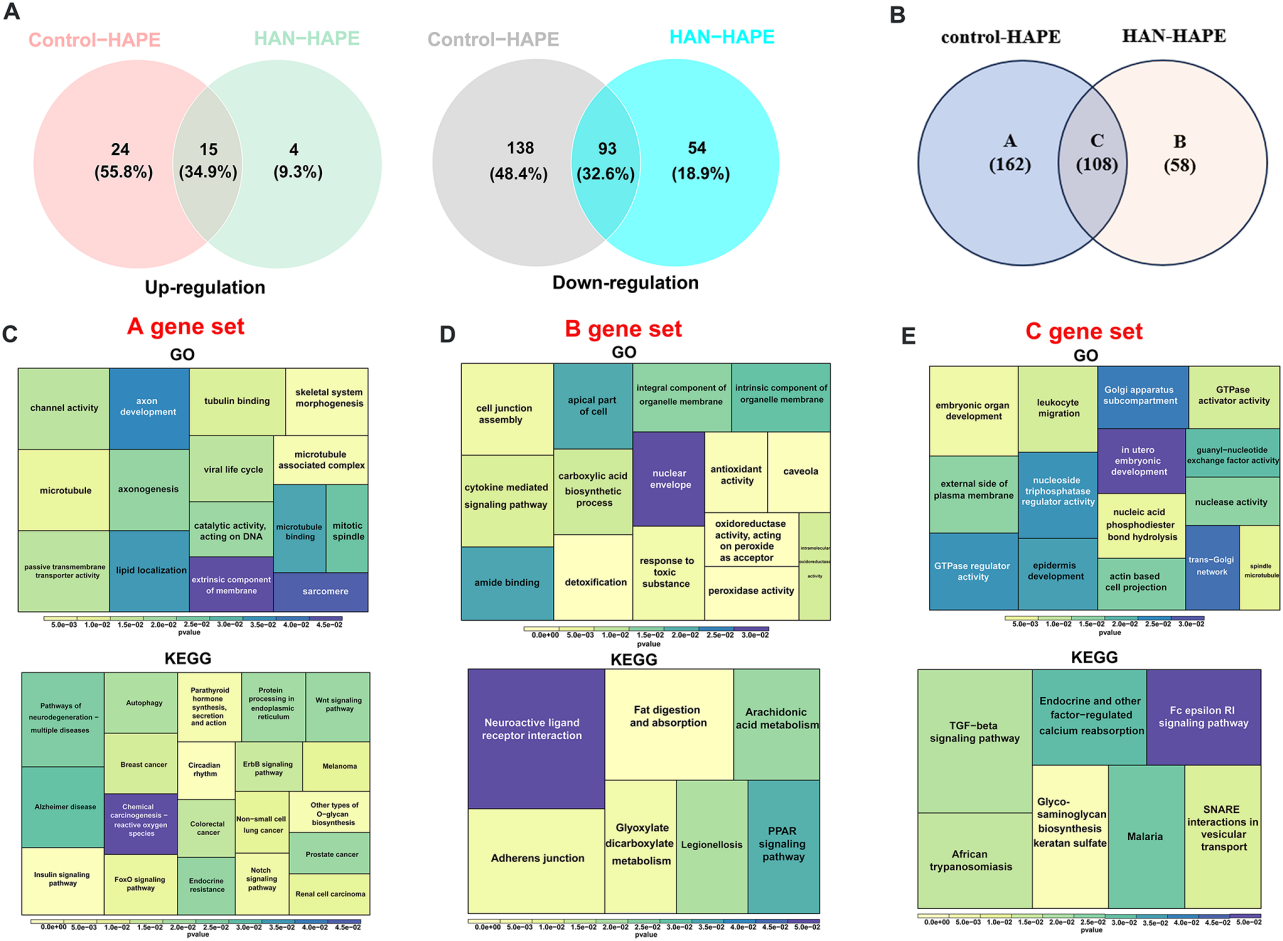
Screening and functional enrichment analysis of common/divergent DEGs. **A**, Venn diagrams showing 15 upregulated DEGs and 93 downregulated DEGs that were altered unanimously in the HAPE group compared with the Control and HAN groups. **B**, Venn diagram showing 162 divergent DEGs that were altered only between the HAPE and Control groups, 58 divergent DEGs that were altered only between the HAPE and HAN groups, and 108 common DEGs that were altered in the HAPE group compared with both the Control and HAN groups. For further analysis, these common/divergent DEGs were classified and marked as gene sets **A**, **B**, and **C**, respectively. **C**-**E**, GO (upper) and KEGG (down) terms of the A gene set (**C**), B gene set (**D**) and C gene set (**E**); the top 5 enriched BP, CC, and MF terms are listed; different squares indicate different pathways, and the color and area of each square are positively correlated with significance and gene count, respectively

Sea-level sojourners and high-altitude natives display distinctive characteristics of physiological responses to hypoxia [9]. To separate the transcript features for HAPE in sojourners and natives, the DEGs between the HAPE and Control groups and between the HAPE and HAN groups were subjected to Venn analysis, with opposite expression trends excluded (Fig. 5A). A total of 108 common DEGs were significantly altered in the HAPE group compared with both the Control and HAN groups, while 162 DEGs were altered only between the HAPE and Control groups, and 58 DEGs were altered only between the HAPE and HAN groups (Fig. 5B). For further analyses, those common/divergent DEGs were classified and marked as gene sets of A (Control-specific), B (HAN-specific) and C (common) (Fig. 5B). Functional enrichment analysis of these common/disparate DEGs revealed that the Control-specific DEGs in the A gene set were enriched mainly in the pathways related to channel activity, axonogenesis, and neurodegenerative disease (Fig. 5C); the HAN-specific DEGs in the B gene set were enriched mainly in the cytokine-mediated signaling pathway and adherens junction (Fig. 5D); and the common DEGs in the C gene set were enriched mainly in embryonic organ development, leukocyte migration and the TGF-beta signaling pathway (Fig. 5E). TGF-ß is a multifunctional cytokine that is particularly critical for embryonic development, wound healing, tissue homeostasis, and immune homeostasis [21]. Thus, these results suggest that the signaling pathways involved in development and immunity are closely associated with HAPE pathogenesis and HAPE susceptibility.

### Identification of hub genes among common/divergent DEGs

**Fig. 6:**
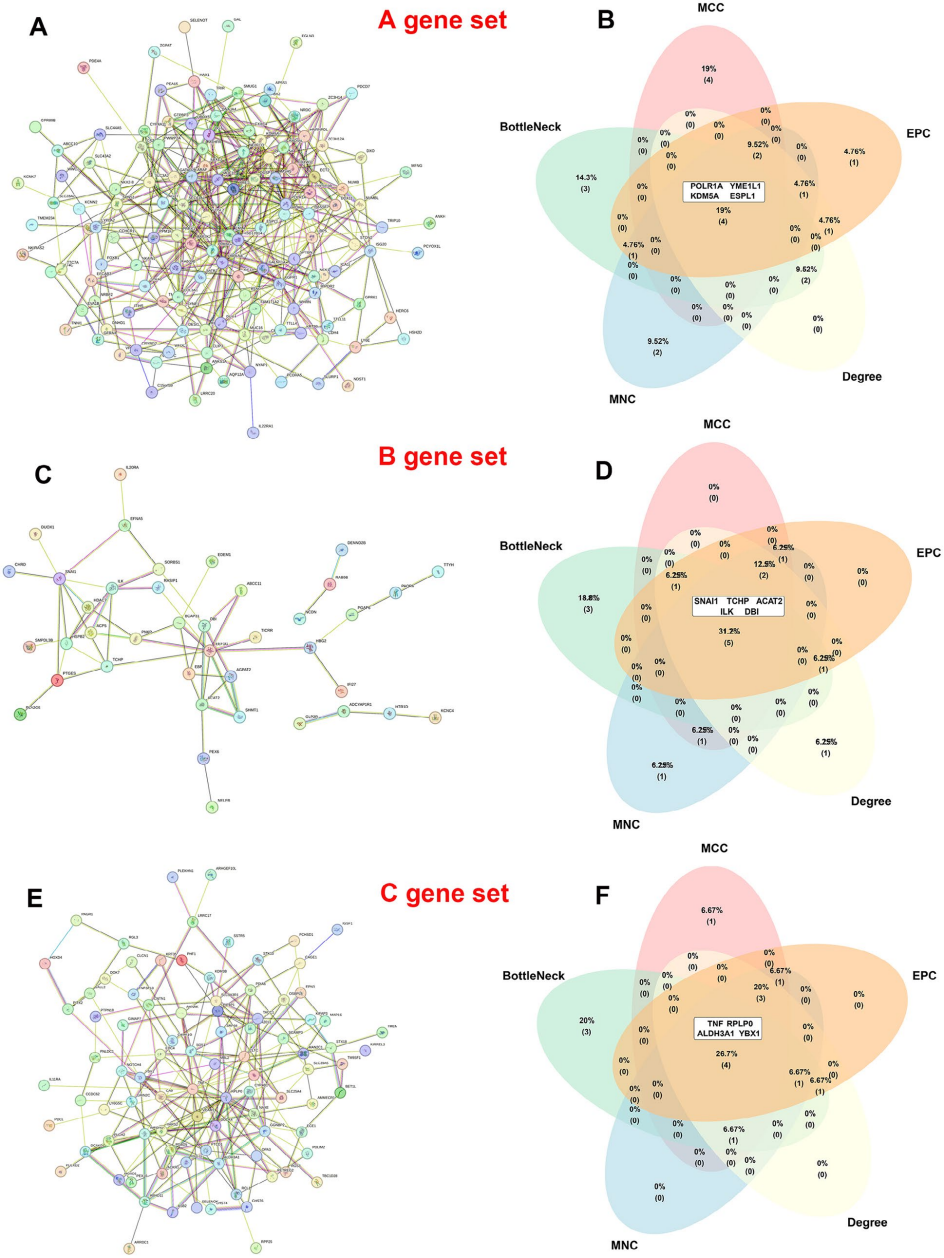
Screening of hub genes in common/divergent DEGs. **A**-**F**, A total of 4, 5 and 4 hub genes were screened in the A/B/C gene sets using STRING and CytoHubba, respectively. PPI networks (**A**, **C** and **E**) and Venn diagrams of 5 algorithms: MCC, MNC, degree, EPC and bottleneck (**B**, **D** and **F**)

PPI networks of these common/divergent DEGs in the A, B, and C gene sets were constructed using STRING and Cytoscape, respectively (Fig. 6A, C and E). Next, based on the five CytoHubba models MCC, MNC, Degree, EPC and BottleNeck, we ranked the top 10 genes within the whole network and performed Venn analysis to obtain the overlapping genes (Fig. 6B, D and F). Finally, through topological analysis algorithms, four genes, POLR1A, YMEIL1, JARIDIA and ESPL1, in the A gene set; five genes, SNAI1, TCHP, ACAT2, ILK and DBI, in the B gene set; and four genes, TNF, RPLP0, ALDH3A1 and YBX1, in the C gene set were identified as the hub genes for HAPE in the sojourners and natives (Fig. 6B, D and F).

### TNFα upregulation and RPLP0 downregulation exhibited significant diagnostic efficacy for HAPE in both sojourners and natives

**Fig. 7:**
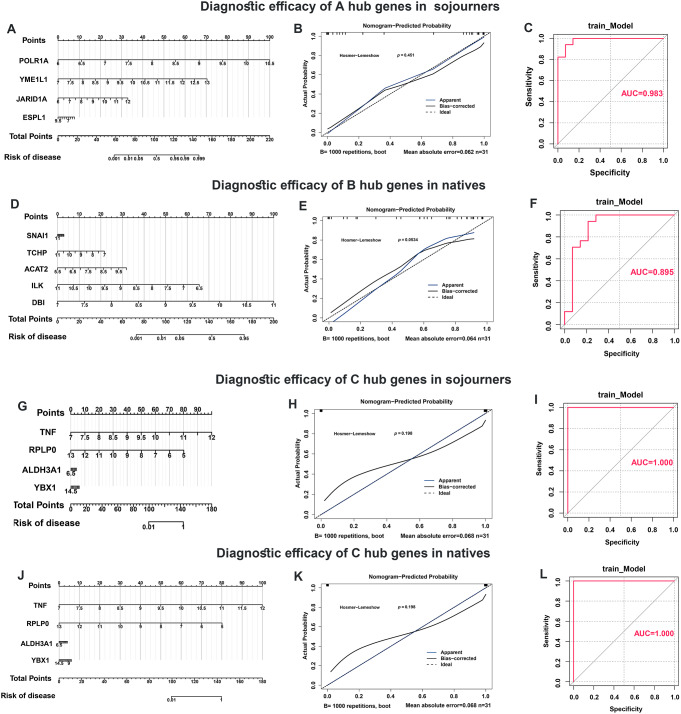
Diagnostic efficacy of the hub genes. **A**-**L**, Diagnostic efficacy of the hub genes was displayed and quantified using nomograms (**A**, **D**, **G** and **J**), calibration curves (**B**, **E**, **H** and **K**) and ROC curves (**C**, **F**, **I** and **L**), respectively. **A**-**C**, Diagnostic efficacy of *POLR1A*, *YME1L1*, *JARID1A* and *ESPL1* (hub genes in the A gene set) in differentiating HAPE patients from acclimatized sojourners. **D**-**F**, Diagnostic efficacy of *SNAI1*, *TCHP*, *ACAT2*, *ILK* and *DBI* (hub genes in the B gene set) in differentiating HAPE patients from adapted natives. **G**-**I**, Diagnostic efficacy of *TNF*, *RPLP0*, *ALDH3A1* and *YBX1* (hub genes in the C gene set) in differentiating HAPE patients from acclimatized sojourners. **J**-**L**, Diagnostic efficacy of *TNF*, *RPLP0*, *ALDH3A1* and *YBX1* (hub genes in the C gene set) in differentiating HAPE patients from adapted natives

Nomograms for HAPE in sojourners and natives were constructed based on the expression of the aforementioned hub genes in the A, B, and C gene sets (Fig. 7A, D, G and J). In these nomograms, the score of each sample was calculated, with a higher score indicating a greater likelihood of HAPE. Calibration curves were used to investigate the predicted probability of the nomogram models, and a slope close to 1 and CIC converging with the trend of the real situation suggested excellent predictive efficacy of these models (Fig. 7B, E, H and K). In addition, ROC curves were used to evaluate the diagnostic sensitivity and specificity of the nomogram models, and the AUC values were as follows: 0.983, 0.895, 1 and 1, all suggesting superior diagnostic efficacy for identifying HAPE patients from acclimatized sojourners and adapted natives (Fig. 7C, F, I and L).

**Fig. 8:**
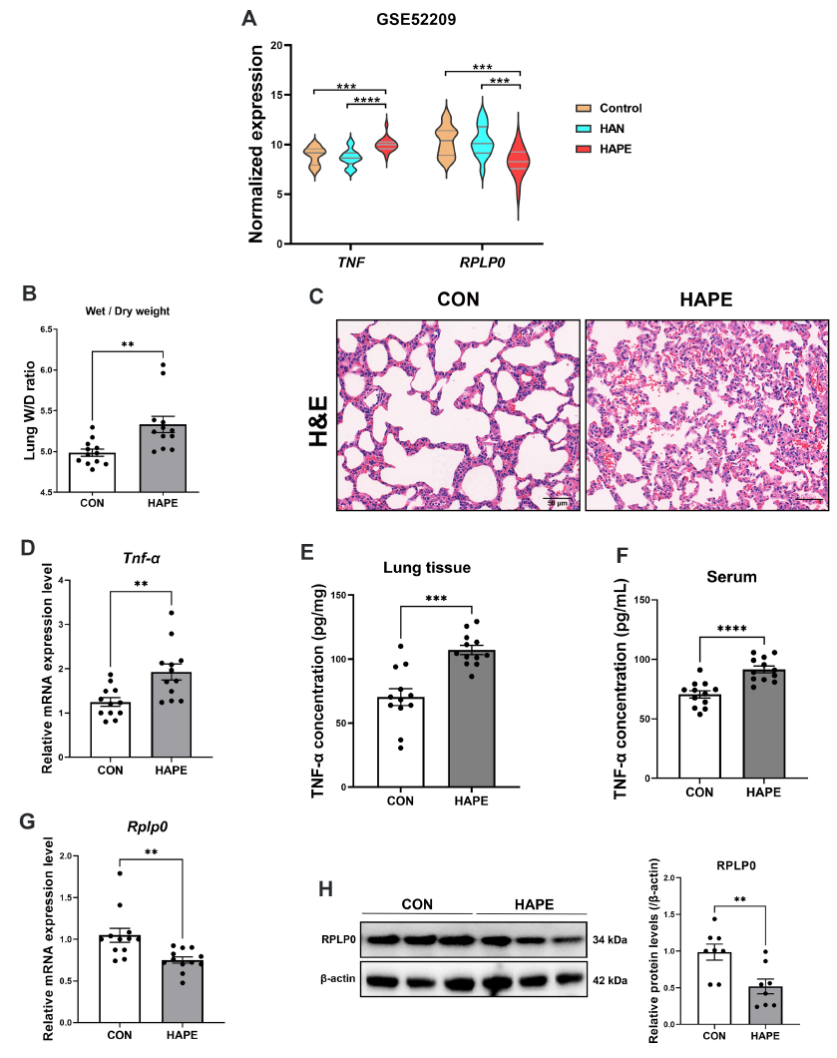
Validation of TNF-α upregulation and RPLP0 downregulation in HAPE rats. **A**, Expression levels of *TNF* and *RPLP0* in GSE52209. **B** and **C**, Rats were subjected to a simulated altitude of 6000 m for 3 days in a hypobaric chamber. The HAPE rat model was verified by assessing the lung wet-to-dry weight ratio (**B**) and histological changes in the lungs using hematoxylin and eosin (HE) staining (**C**) (*n* = 12 for each group; scale bars: 50 μm). **D**, Relative mRNA expression level of *Tnf-α* in the lung tissue of HAPE rats, as determined by RT‒qPCR analysis (*n* = 12 for each group). **E** and **F**, Protein levels of TNF-α in the lung tissue (**E**) and serum (**F**) of HAPE rats were measured using ELISA (*n* = 12 for each group). **G**, Relative mRNA expression level of *Rplp0* in the lung tissue of HAPE rats, as determined by RT‒qPCR analysis (*n* = 12 for each group). **H**, Relative protein level of RPLP0 in the lung tissue of HAPE rats, as determined by western blotting analysis (*n* = 8 for each group). Continuous variables with a normal distribution are presented as means ± SEM. Significance was determined by ordinary one-way ANOVA followed by Tukey’s multiple comparisons (**A**) and 2-tailed unpaired Student’s t test with Welch’s correction (**B** and **D**-**H**). *P < 0.05, **P < 0.01, *** *P* < 0.001, **** *P* < 0.0001 versus the control

In the nomograms, the line segment length represents the degree of contribution of each hub gene to the outcome event, and among the four hub genes in the C gene set, TNF and RPLP0 showed high efficacy in predicting and diagnosing HAPE both in sojourners and natives (Fig. 7G and J). In addition, TNF expression was significantly increased, whereas RPLP0 expression was significantly decreased in the HAPE group compared with both the acclimatized sojourners and adapted natives (Fig. 8A), suggesting potentially critical implications of TNF and RPLP0 in the susceptibility to HAPE.

### Validation of TNF-α upregulation and RPLP0 downregulation in HAPE rats

To establish the HAPE animal model, the rats were subjected to a simulated altitude of 6000 m (the barometric pressure is about 353.88 mmHg and oxygen partial pressure is about 74.0 mmHg) for 3 days in a hypobaric chamber, and the lung wet-to-dry weight ratio and histological changes in the lungs were examined as previously reported [16]. As shown in Fig. 8B, after 3 days of exposure to a simulated altitude of 6000 m, the wet-to-dry weight ratio of rat lung tissues significantly increased (5.333 ± 0.09876 for HAPE versus 4.985 ± 0.04353 for the control). Hematoxylin and eosin (HE) staining also revealed obvious ultrastructural damage, including alveolar edema and hemorrhage, and lung interstitial thickening and deformation, in the rat lung tissues (Fig. 8C), suggesting that the rat model of HAPE was constructed successfully. RT-qPCR analysis revealed that TNF-α mRNA expression significantly increased (1.927 ± 0.1798 for HAPE versus 1.248 ± 0.09936 for the control) and that RPLP0 expression significantly decreased (0.7506 ± 0.03789 for HAPE versus 1.048 ± 0.08392 for the control) in the lung tissues of HAPE rats (Fig. 8D and G). ELISA revealed that the protein level of TNF-α significantly increased in the lung tissues (107.1 ± 3.636 for HAPE versus 70.28 ± 6.569 for the control) and serum (91.53 ± 2.865 for HAPE versus 70.53 ± 3.064 for the control) of HAPE rats (Fig. 8E and F). Western blotting analysis revealed that the protein level of RPLP0 significantly decreased (0.9857 ± 0.1095 for HAPE versus 0.5180 ± 0.1001 for the control) in the lung tissues of HAPE rats (Fig. 8H). These results were consistent with and verified the results of bioinformatic analysis in GSE52209.

### Pulmonary EC apoptosis and endothelial permeability are increased in the lung tissues of HAPE rats

**Fig. 9:**
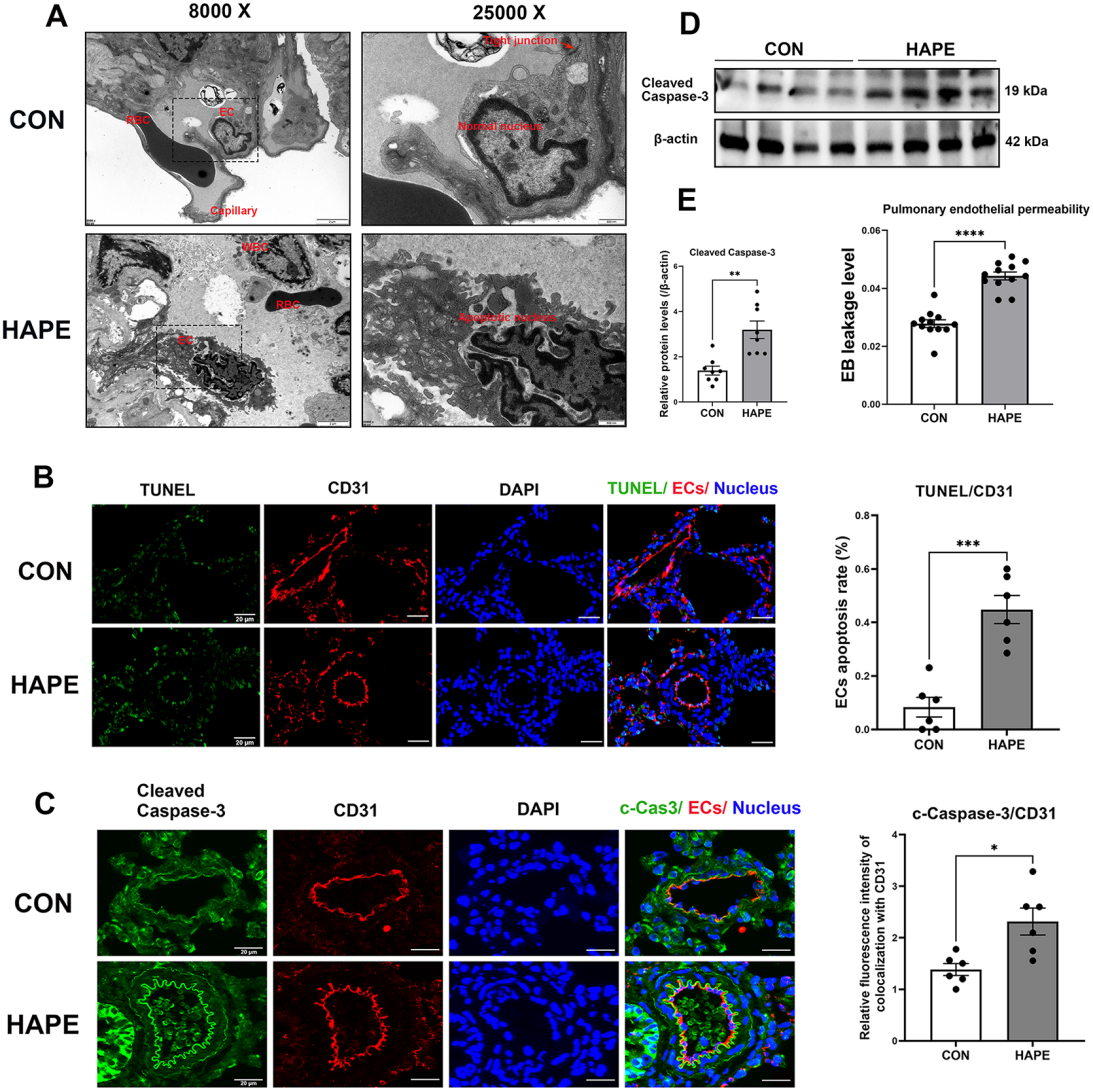
Pulmonary EC apoptosis and vasculature permeability were enhanced in HAPE rats. **A**, Ultrastructural changes in HAPE rat lung tissues detected by TEM; scale bars: 2 μm (left) and 500 nm (right). **B**, Rat lung tissues were subjected to TUNEL (green) and CD31 (EC indicator, red) staining using in situ immunofluorescence. EC apoptosis rates were assessed by quantitative comparison of TUNEL^+^ CD31^+^ cells (*n* = 6 for each group; scale bars: 20 μm). **C**, Rat lung tissues were stained for cleaved caspase-3 (green) and CD31 (red) using in situ immunofluorescence. The relative protein level of cleaved caspase-3 in the lung ECs was assessed by quantitative comparison of the fluorescence intensity of cleaved caspase-3^+^ CD31^+^ cells (*n* = 6 for each group; scale bars: 20 μm). **D**, Relative protein level of cleaved caspase-3 in the lung tissues of HAPE rats, as determined by western blotting analysis (*n* = 8 for each group). **E**, Pulmonary endothelial permeability was examined by measuring Evans blue dye leakage levels in lung tissues (*n* = 12 for each group). Continuous variables with a normal distribution are presented as the means ± SEMs. Significance was determined by 2-tailed unpaired Student’s t test with Welch’s correction (**B**-**E**). *P < 0.05, **P < 0.01, *** *P* < 0.001, **** *P* < 0.0001 versus the control

HAPE is a high-permeability pulmonary edema characterized by exaggerated vascular leakage, and it has been reported that EC apoptosis plays an important role in the regulation of endothelial permeability [22–24]. In the present study, using transmission electron microscopy (TEM), we observed significant destruction of the capillary endothelium and tight junctions, as well as signs of apoptosis with nuclear chromatin condensation and fragmentation and perinuclear space broadening in the ECs of HAPE rat lung tissues (Fig. 9A). Next, TUNEL staining (Fig. 9B), along with double-immunofluorescence staining and immunoblotting of apoptosis-related proteins (cleaved caspase-3) (Fig. 9C and D), were conducted to assess EC apoptosis in rat lung tissues. The EC apoptosis rate (44.84% ± 5.255% for HAPE versus 8.319% ± 3.685% for the control) and expression of cleaved caspase-3 in the endothelium (2.314 ± 0.2616 for HAPE versus 1.384 ± 0.1153 for the control in Fig. 9C.19 ± 0.3883 for HAPE versus 1.389 ± 0.1964 for control in Fig. 9D) were both significantly increased in HAPE rats. Lung endothelial permeability was examined by measuring Evans blue dye accumulation and leakage in lung tissues, and the results suggested increased endothelial permeability and vascular leakage (0.04428 ± 0.001375 for HAPE versus 0.02781 ± 0.001339 for the control) in the lung tissues of HAPE rats (Fig. 9E). These results indicated the activation of pulmonary EC apoptosis and high endothelial permeability in the lung tissues of HAPE rats.

### The addition of TNF-α and RPLP0 knockdown induced EC apoptosis and increased endothelial permeability

**Fig. 10:**
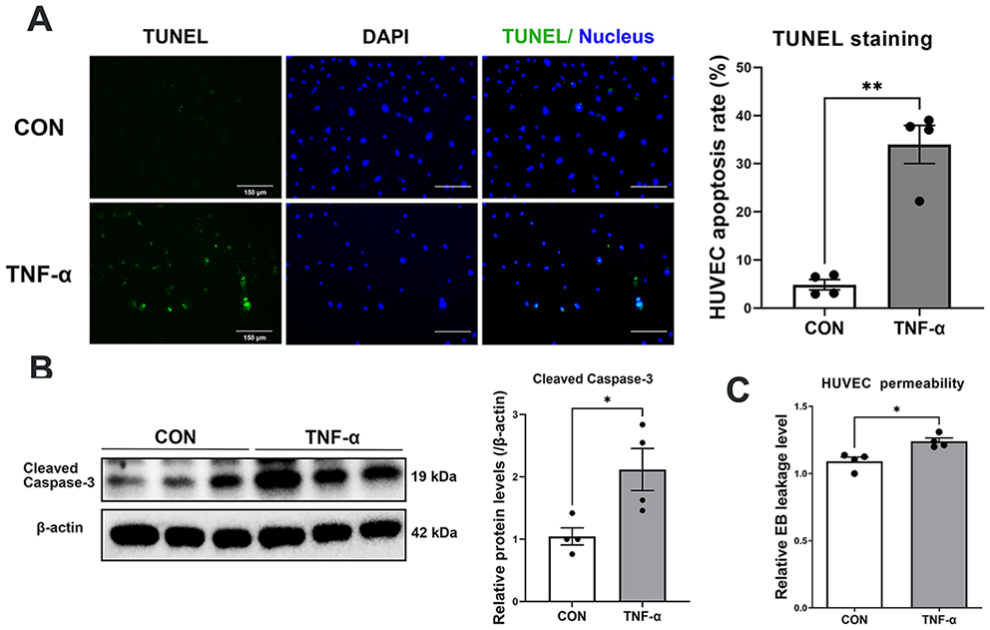
The addition of TNF-α activated apoptosis signaling and increased endothelial permeability in HUVECs. **A**-**C**, HUVECs were treated with 40 ng/ml TNF-α for 48 h. **A**, Cell apoptosis was examined by the TUNEL assay (*n* = 4 for each group). **B**, Relative protein level of cleaved caspase-3 in HUVECs, as determined by western blotting analysis (*n* = 4 for each group). **D**, HUVEC permeability was examined by measuring the Evans blue leakage level using a transwell system (*n* = 4 for each group). Continuous variables with a normal distribution are presented as the means ± SEMs. Significance was determined by 2-tailed unpaired Student’s t test with Welch’s correction (A-C). *P < 0.05, **P < 0.01, *** *P* < 0.001, **** *P* < 0.0001 versus the control

TNF-α can directly activate apoptosis signaling [25]. In the in vitro experiments, cultured HUVECs were treated with 40 ng/ml of TNF-α for 24 h, and TUNEL staining and western blotting analysis revealed that both the apoptosis rate (34% ± 3.947% for TNF-α versus 4.854% ± 1.056% for the control) and the expression of cleaved caspase-3 (2.119 ± 0.3389 for TNF-α versus 1.045 ± 0.1369 for the control) in HUVECs were significantly increased by stimulation with TNF-α (Fig. 10A and B). Next, HUVECs were seeded in a transwell chamber to form an endothelial monolayer in vitro. Endothelial permeability was assessed by measuring the amount of Evans blue dye leakage from the upper chamber to the lower chamber, and the results suggested that the addition of TNF-α significantly increased endothelial permeability (1.24 ± 0.02452 for TNF-α versus 1.091 ± 0.03113 for the control) in vitro (Fig. 10C).

**Fig. 11:**
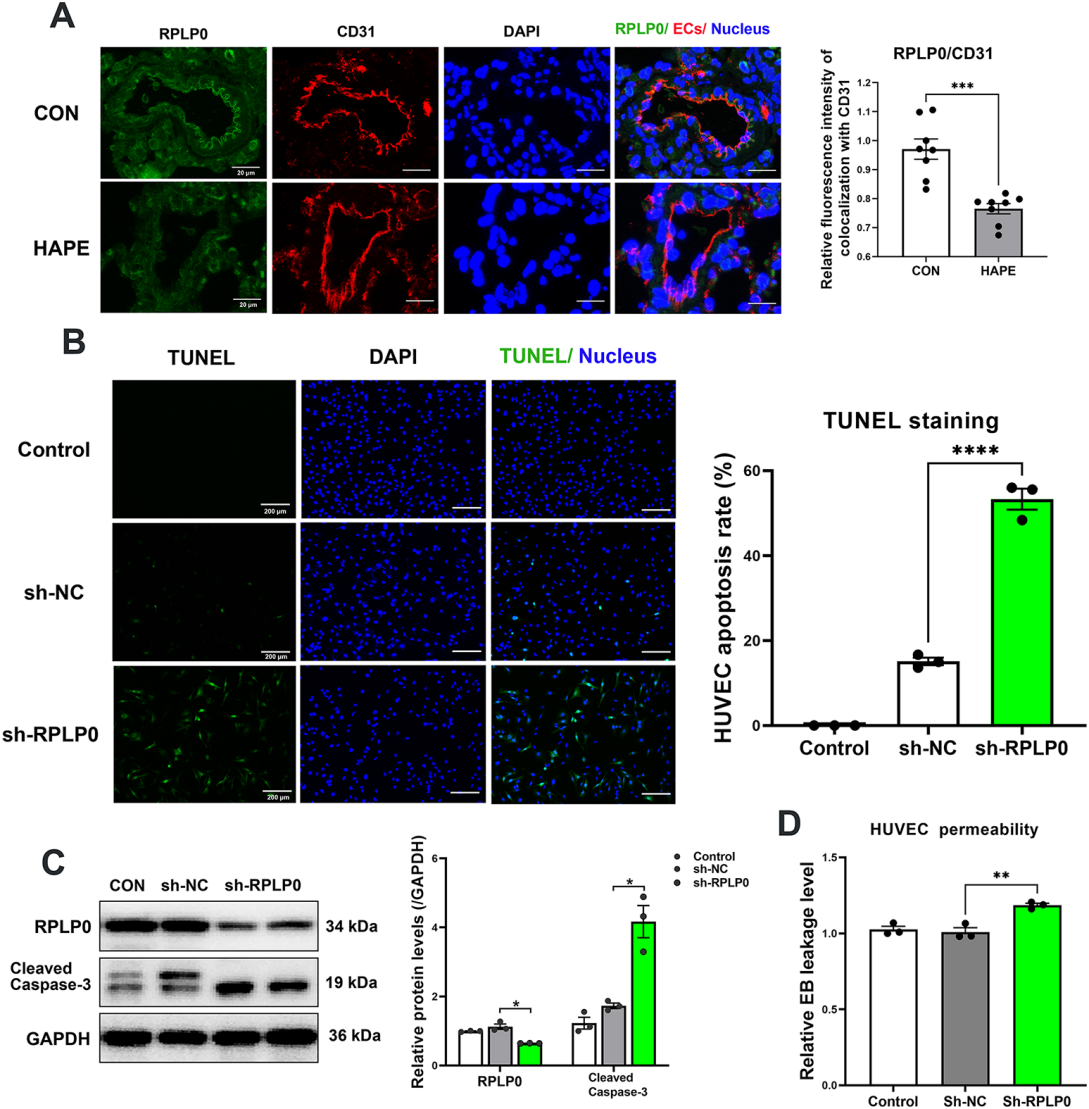
RPLP0 knockdown activated apoptosis signaling and increased endothelial permeability in HUVECs. **A**, Rat lung tissues were stained for RPLP0 (green) and CD31 (EC indicator, red) by in situ immunofluorescence. The relative protein level of RPLP0 in the lung ECs was assessed by quantitative comparison of the fluorescence intensity of RPLP0^+^ CD31^+^ cells (*n* = 8 for each group; scale bars: 20 μm). **B**-**D**, RPLP0 was knocked down in cultured HUVECs by lentiviral transduction with RPLP0-shRNA. **B**, Cell apoptosis in RPLPP0-knockdown HUVECs was examined using the TUNEL assay (*n* = 3 for each group). **C**, Relative protein level of cleaved caspase-3 in RPLPP0-knockdown HUVECs, as determined by western blot analysis (*n* = 3 for each group). **D**, HUVEC permeability was examined by measuring the Evans blue leakage level using a transwell system (*n* = 3 for each group). Continuous variables with a normal distribution are presented as the means ± SEMs. Significance was determined by 2-tailed unpaired Student’s t test with Welch’s correction (**A**) and ordinary one-way ANOVA followed by Tukey’s multiple comparisons (**B**-**D**). *P < 0.05, **P < 0.01, *** *P* < 0.001, **** *P* < 0.0001 versus the control or sh-NC

Double-immunofluorescence staining of RPLP0 and CD31 revealed that RPLP0 expression significantly decreased (0.7650 ± 0.0176 for HAPE versus 0.9706 ± 0.035 for the control) in the pulmonary endothelium of HAPE rats (Fig. 11A). Next, RPLP0 expression was silenced in HUVECs using lentiviruses carrying RPLP0-specific shRNAs (0.6436 ± 0.002791 for sh-RPLP0 versus 1.125 ± 0.07545 for sh-NC). TUNEL staining and western blotting analysis revealed that both the HUVEC apoptosis rate (53.31% ± 2.467% for sh-RPLP0 versus 15.1% ± 0.8762% for sh-NC) and cleaved caspase-3 expression (4.169 ± 0.4659 for sh-RPLP0 versus 1.73 ± 0.07924 for sh-NC) were significantly increased in the RPLP0 shRNA-HUVECs (Fig. 11B and C). Next, HUVECs infected with RPLP0 shRNA were seeded to establish the endothelial monolayer, and Evans blue dye leakage was measured using a transwell system. When RPLP0 was knocked down, the leakage of Evans blue dye through the HUVEC monolayer markedly increased (1.185 ± 0.01279 for sh-RPLP0 versus 1.009 ± 0.02817 for sh-NC), suggesting increased endothelial permeability (Fig. 11D). These results indicate that both TNF-α stimulation and RPLP0 downregulation can activate EC apoptosis signaling and thus increase endothelial permeability.

### Prediction of competing endogenous RNAs (ceRNAs) and drugs targeting hub genes

**Fig. 12:**
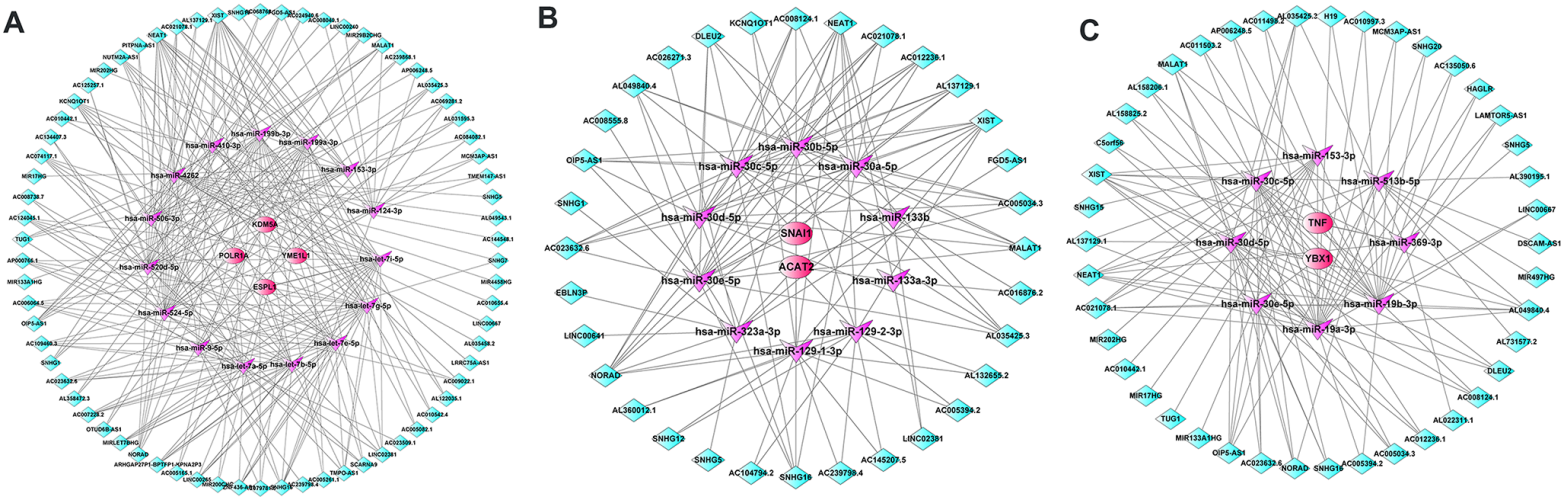
Construction of the ceRNA network targeting hub genes. **A**-**C**, ceRNA regulatory networks were constructed targeting hub genes in the **A**, **B** and **C** gene sets, respectively. The red circles represent the hub genes, the purple stars represent miRNAs, and the green rectangles represent lncRNAs. The black lines indicate the lncRNA-miRNA-mRNA interactions. ceRNAs, competing endogenous RNAs; miRNAs, microRNAs; lncRNAs, long noncoding RNAs

By targeting the hub genes in the A/B/C gene sets, we predicted the mRNA-miRNA interaction pairs using the Elmmo database and selected the 5 miRNAs with the highest binding scores to further predict the miRNA-lncRNA interactions using the starBase database. Finally, ceRNA networks of mRNAs-miRNAs-lncRNAs were constructed and visualized using Cytoscape (Fig. 12A and C).

**Fig. 13:**
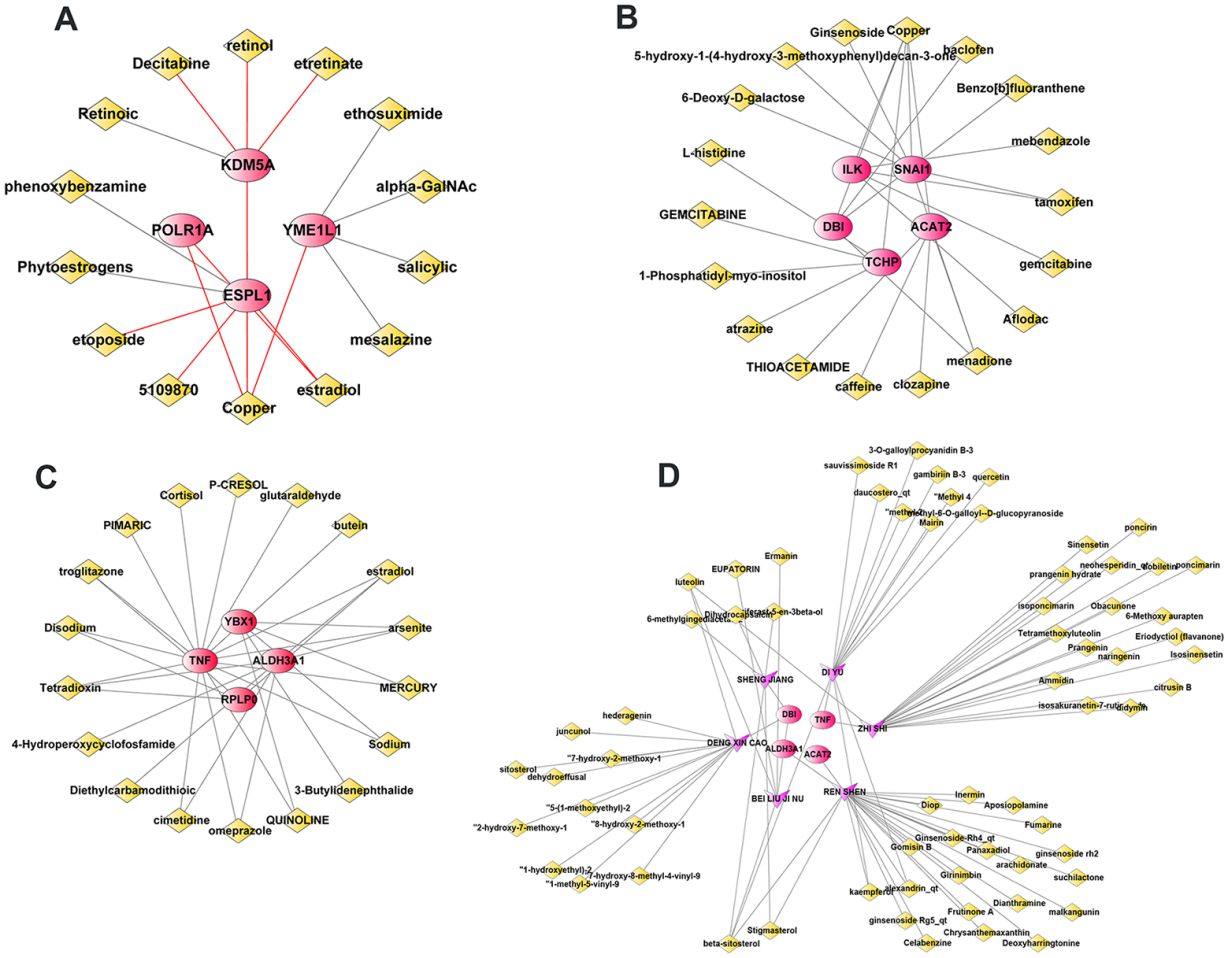
Prediction of drugs and compounds targeting hub genes. **A-C**, Candidate drugs were predicted to target the hub genes in the **A**, **B** and **C** gene sets, respectively. **D**, Candidate compounds of traditional Chinese medicines (TCMs) were predicted to target hub genes

Using the DSigDB database, potentially effective drugs targeting the hub genes were predicted (Fig. 13A and C). In addition, using the ETC and TCMSP databases, candidate compounds of traditional Chinese medicines (TCMs) that target hub genes were also predicted (Fig. 13D). Finally, networks of drug-gene pairs and TCM-compound-gene pairs were established and visualized using Cytoscape.

## Discussion

HAPE is a fatal threat for people who ascend rapidly to high altitudes but without sufficient acclimatization [26]. The incidence of HAPE has individual susceptibility tendencies, and high-altitude natives and sojourners with sufficient acclimatized responses can accommodate hypobaric hypoxic stress well and are insusceptible to HAPE. However, the physiological traits and mechanisms of HAPE susceptibility differ between acclimatized sojourners and adapted natives, which results from evolved genetic specializations [9]. In the present study, the GSE52209 dataset was analyzed to compare the gene expression profiles of HAPE patients with acclimatized sojourners and adapted natives. The analyses of DEG functional enrichment and immune infiltration suggested that the molecular basis and immune involvement of HAPE susceptibility were different in the acclimatization and adaptation processes.

Clarifying the distinctive molecular basis of acclimatization and adaptation processes in sojourners and natives could provide a more comprehensive understanding of HAPE susceptibility. Thus, the two groups of DEGs were overlapped by Venn analysis to screen the common and divergent DEGs for HAPE between acclimatized sojourners and adapted natives. Functional analysis revealed that the DEGs that were altered only between HAPE and acclimatized sojourner groups were enriched in channel activity, the DEGs that were altered only between HAPE and adapted native groups were enriched in cytokine and adherens pathways, and the common DEGs that overlapped between acclimatized sojourner and adapted native groups were enriched in the pathways of development and immunity [21]. These findings indicate that channel activity is involved in the acclimatization mechanism of sojourner insusceptibility to HAPE and that the signaling pathways of development and immunity are involved in both the acclimatization and adaptation mechanisms of sojourner and native insusceptibility to HAPE. The pathophysiology of HAPE is currently attributed to heterogeneous hypoxic pulmonary vasoconstriction and the subsequent intercellular mechanisms of increased endothelial permeability, inflammation, and damaged fluid reabsorption capacity and matrix architecture [26]. Channel activity plays a role in regulating hypoxic pulmonary vasoconstriction [27], and cytokines promote lung endothelial permeability and leakage [28]. A number of comparative studies have reported the different regulatory plasticity of physiological systems in response to hypoxia between high-altitude natives and sea-level sojourners [9, 10]. Our findings that the enrichment of channel activity in sojourners but not in natives are consistent with their distinctive physiological manifestations, in which excessive and uneven hypoxic pulmonary vasoconstriction triggers HAPE in sojourners, whereas high-altitude natives show attenuated hypoxic pulmonary vasoconstriction [9, 26]. In addition, the enrichment of development and immunity pathways in both sojourners and natives suggests that some phenotypic features, such as increased lung volume and capacity, abundant pulmonary capillaries and increased nitric oxide metabolites, along with inflammatory signaling, are critically implicated in both acclimatization and adaptation mechanisms at high altitudes. Variations in or abnormalities in development and immunity signaling may increase individual susceptibility to HAPE.

Based on the topological analysis algorithms, hub genes were subsequently screened out in these common/divergent DEGs, and nomogram models revealed that TNF-α and RPLP0 were critically implicated in both acclimatization and adaptation mechanisms and exhibited high diagnostic efficiency for HAPE susceptibility in both sojourners and natives. Apoptotic endothelial cell death critically disturbs the integrity of the endothelial monolayer and thereby exacerbates capillary leakage and pulmonary edema [29, 30]. As a pro-apoptotic inflammatory cytokine, TNF-α can directly activate at least 5 different types of cellular signaling, including NF-κB, apoptosis, ERK, p38 and JNK-MAPK, and play a central role in orchestrating mammalian inflammatory responses either directly by inducing inflammatory gene expression or indirectly by triggering cell death via apoptosis, necroptosis, or pyroptosis to activate innate immune receptors on neighboring cells [25, 31]. In particular, by inducing the apoptosis of epithelial and endothelial cells, TNF-α disrupts barrier integrity and vascular integrity to initiate pathogen-associated molecular pattern (PAMP)-mediated inflammatory signaling and tissue injury [25, 32]. It has generally been reported that hypoxia activates inflammatory signaling and that HAPE patients present significant increases in the levels of inflammatory cytokines, including TNF-α, which is consistent with our present findings [28]. Ribosomal protein P0 (RPLP0) is a component of the ribosome 60 S subunit located in the cytoplasm and nucleus, which appears to be involved not only in protein synthesis but also in transcriptional processes, DNA repair, cell development, cell apoptosis, and tumorigenesis [33, 34]. High expression levels of RPLP0 have been detected in many tumors [34], and depletion of RPLP0 can lead to apoptosis and cell cycle arrest in many cancer cells [33, 34]. In addition, evidence suggests that RPLP0 is highly expressed in vascular endothelial cells [35]. In the present study, through an array of in vivo and in vitro experiments, we confirmed the upregulation of TNF-α and downregulation of RPLP0 in the lung tissues of HAPE rats and further revealed that the addition of TNF-α and the knockdown of RPLP0 could activate EC apoptosis signaling to increase endothelial permeability during HAPE pathogenesis. Previous studies reported that β2 microglobulin (B2M) could act as a clinical biomarker for the early diagnosis and therapeutic evaluation of HAPE, and its decrease may be related to the reduced immune ability of Major Histocompatibility Complex Class I (MCH I) in antigen submission [13]. However, this study did not describe from which population, sojourners or natives, they drew their conclusions. In addition, another study reported that the HAPE prevalence in Andean highlanders was strongly related to the increase in the mean corpuscular hemoglobin concentration (MCHC), but its specific molecular basis has not been explored [36]. Our present findings not only confirmed the diagnostic value of TNF-α upregulation and RPLP0 downregulation as specific biomarkers for HAPE susceptibility, but also further clarified the specific molecular mechanism by which TNF-α upregulation and RPLP0 downregulation increase HAPE susceptibility by driving EC apoptosis and enhancing endothelial permeability. Furthermore, to inspire new treatment strategies, networks of ceRNAs and drugs targeting these hub genes were also predicted and constructed in our present study.

However, some limitations of the study warrant further consideration. First, although we validated hub gene expression in the HAPE rat model, further experiments using human samples are still needed to confirm our findings because of the inherent limitations of bioinformatics techniques. Second, since our data were sourced from a public database and the human subjects/rats included were all male, the covariates of sex, age and comorbidities were not considered. Further clinical studies and higher levels of evidence are needed. In addition, the heterogeneity of blood samples may strongly affect gene transcript abundance, making it difficult to identify whether the increased total expression is due to gene overexpression or only having more cells of a given subset in the sample; hence, there is a limitation in interpreting the immune infiltration results of cell subset alterations [37].

## Conclusion

In summary, the differential gene expression profiles of HAPE patients between acclimatized sojourners and adapted natives revealed the different molecular basis of acclimatization and adaptation in sojourners and native individuals at high altitudes and provided a novel perspective for understanding HAPE pathogenesis and susceptibility. The identification and validation of TNF-α and RPLP0 as hub genes that have high diagnostic efficacy for HAPE in both sojourners and natives and the subsequent mechanism exploration revealed that the upregulation of TNF-α and downregulation of RPLP0 activated EC apoptosis signaling to increase endothelial permeability in HAPE pathogenesis confirmed the role of TNF-α and RPLP0 as shared and critical biomarkers of HAPE pathogenesis and susceptibility during the acclimatization/adaptation/maladaptation processes.

## Electronic supplementary material

Below is the link to the electronic supplementary material.

Supplementary Material 1

## Data Availability

No datasets were generated or analysed during the current study.

## Abbreviations

HAPE: High-altitude pulmonary edema
ECs: Endothelial cells
HUVEC: Human primary umbilical vein endothelial cell
HAN: High-altitude natives
TNF-α: Tumor necrosis factor-alpha
RPLP0: Ribosomal protein lateral stalk subunit P0
DEGs: Differentially expressed genes
GO: Gene Ontology
KEGG: Kyoto Encyclopedia of Genes and Genomes
PPI: Protein–protein interaction
TUNEL: TdT-mediated dUTP-biotin nick end labeling
ROC: Receiver operating characteristic
ceRNA: Competing endogenous RNA
TCM: Traditional Chinese Medicine
