# Supplementary material for: TNF-α and RPLP0 drive the apoptosis of endothelial cells and increase susceptibility to high-altitude pulmonary edema
Source: Apoptosis. 2024 Aug 7;29(9-10):1600–18. doi: 10.1007/s10495-024-02005-9 (PMC11416372; doi:10.1007/s10495-024-02005-9)
Supplement: Supplementary file 1 — Supplementary Material 1 [file 10495_2024_2005_MOESM1_ESM.docx]

**Supplemental Tables:**

Table S1. Sequences of *RPLP0* shRNAs

| **shRNAs** | **Forward** | **Reverse** |
| --- | --- | --- |
| shRPLP0-296 | CACCGGGACATGTTGCTGGCCAATA- CGAATATTGGCCAGCAACATGTCCC | CACCGGGACATGTTGCTGGCCAATA-  CGAATATTGGCCAGCAACATGTCCC |
| shRPLP0-765 | CACCGGATTACACCTTCCCACTTGCC- GAAGCAAGTGGGAAGGTGTAATCC | AAAAGGATTACACCTTCCCACTTGC-  TTCGGCAAGTGGGAAGGTGTAATCC |
| shRPLP0-921 | CACCGGACGAGGATATGGGATTTGGC-GAACCAAATCCCATATCCTCGTCC | AAAAGGACGAGGATATGGGATTTGGTT-CGCCAAATCCCATATCCTCGTCC |

Table S2. Primers for RT-qPCR

| \| **Gene** \| **Species** \| **Forward** \| **Reverse** \| \| --- \| --- \| --- \| --- \| \| ACTB \| Rat \| GGACCTGACAGACTACCTCA \| GTTGCCAATAGTGATGACCT \| \| RPLP0 \| Rat \| CCCTTCTCCTTCGGGCTGAT \| TGAGGCAACAGTCGGGTAGC \| \| TNF \| Rat \| TTGTCTACTCCCAGGTTCTCT \| GAGGTTGACTTTCTCCTGGTATG \| | **Homo sapiens** | **TCACCATGGATGATGATATCGC** | **ATAGGAATCCTTCTGACCCATGC** |
| --- | --- | --- | --- | --- | --- | --- | --- | --- | --- | --- | --- | --- | --- | --- | --- | --- | --- | --- | --- |

Table S3. Original data of endothelial permeability detection in HAPE rat

| Groups  (Control/HAPE) | Evans blue dye absorbance at 620 nm | Protein concentration（ug/ul) | Permeability  (EB/Protein) |
| --- | --- | --- | --- |
| Control-1 | 0.373166667 | 14.4335576 | 0.025854102 |
| Control-2 | 0.3081 | 17.71947115 | 0.017387652 |
| Control-3 | 0.406566667 | 14.6041089 | 0.027839197 |
| Control-4 | 0.3069 | 9.8685093 | 0.031098922 |
| Control-5 | 0.3991 | 13.31750475 | 0.029968076 |
| Control-6 | 0.1688 | 5.9532988 | 0.028354028 |
| Control-7 | 0.13155 | 4.89077665 | 0.026897569 |
| Control-8 | 0.1292 | 4.99161355 | 0.025883414 |
| Control-9 | 0.1547 | 5.96139065 | 0.025950321 |
| Control-10 | 0.1661 | 6.0317275 | 0.027537716 |
| Control-11 | 0.17705 | 6.06471735 | 0.029193446 |
| Control-12 | 0.2634 | 7.1110558 | 0.037040913 |
| HAPE-1 | 0.2197 | 5.9458294 | 0.03695027 |
| HAPE-2 | 0.2747 | 6.55520795 | 0.041905612 |
| HAPE-3 | 0.26705 | 6.26763605 | 0.042607771 |
| HAPE-4 | 0.3528 | 6.92743305 | 0.050927955 |
| HAPE-5 | 0.3261 | 7.5617096 | 0.043125168 |
| HAPE-6 | 0.35695 | 7.53245445 | 0.047388272 |
| HAPE-7 | 0.26725 | 7.41294405 | 0.036051803 |
| HAPE-8 | 0.2971 | 6.62990195 | 0.044812126 |
| HAPE-9 | 0.3128 | 6.75937155 | 0.046276491 |
| HAPE-10 | 0.3264 | 7.37435215 | 0.044261515 |
| HAPE-11 | 0.34715 | 7.0351169 | 0.049345307 |
| HAPE-12 | 0.34985 | 7.1894845 | 0.048661347 |
